# Mobile-BAT—A Novel Ultra-Low Power Wildlife Tracking System

**DOI:** 10.3390/s23115236

**Published:** 2023-05-31

**Authors:** Stefan Erhardt, Martin Koch, Andreas Kiefer, Michael Veith, Robert Weigel, Alexander Koelpin

**Affiliations:** 1 Institute of High Frequency Technology, Hamburg University of Technology, Denickestraße 22, 21073 Hamburg, Germany; stefan.erhardt@fau.de; 2 Institute for Electronics Engineering, Friedrich-Alexander-Universität Erlangen-Nürnberg, Cauerstraße 9, 91058 Erlangen, Germany; robert.weigel@fau.de; 3 Department of Biogeography, University of Trier, Universitätsring 15, 54286 Trier, Germany; kochmartinp@gmail.com (M.K.); kiwikiefera@gmail.com (A.K.); veith@uni-trier.de (M.V.)

**Keywords:** GSM, mobile communication, telemetry, low-power electronics, radio propagation, wireless sensor networks, bats, movement ecology, wildlife tracking

## Abstract

We introduce a novel ultra-low power system for tracking animal movements over long periods with an unprecedented high-temporal-resolution. The localization principle is based on the detection of cellular base stations using a miniaturized software-defined radio, weighing 2.0 g, including the battery, and having a size equivalent to two stacked 1-euro cent coins. Therefore, the system is small and lightweight enough to be deployed on small, wide-ranging, or migrating animals, such as European bats, for movement analysis with an unprecedented spatiotemporal resolution. The position estimation relies on a post-processing probabilistic RF pattern-matching method based on the acquired base stations and power levels. In several field tests, the system has been successfully verified, and a run-time of close to one year has been demonstrated.

## 1. Introduction

Studying the movements of free-ranging animals is important for understanding their behaviors, spatial ecology, and energetics [1]. Animals carry out movements for various reasons, such as foraging, territory defense, and seasonal migration. However, in a changing landscape, habitat connectivity can be reduced by deforestation, agriculture, urbanization, and other anthropogenic activities [2]; moreover, functional connectivity at the landscape level can become lost. Therefore, tracking animal routes provides knowledge to predict how they respond to alterations in their environments, e.g., landscape or climate changes [3]. This is crucial for the development of successful conservation strategies.

However, the movements of most species in the wilderness are not yet well understood. This is particularly true for species that are lightweight and move over long distances, such as small birds, bats, or insects [4,5,6]. The lack of such knowledge is known as the “small animal problem”, regarding the limitations of the load-carrying abilities of small, lightweight animals [4]. Unfortunately, most currently available tracking devices are either too heavy to be attached to small animals or have very limited functionality.

VHF (very high frequency) transmitters, geolocators, and (more recently) GPS loggers, have been used to track small lightweight animals [7,8,9]. When attached, the device should not influence the behavior of the animals or reduce their probability of survival. Therefore, the transmitter weight is limited to a fraction of its own weight [10]. Hence, the use of a given device is a compromise between spatiotemporal resolution and longevity, the latter being the battery capacity constraint, which is directly correlated to the total weight of the sensor. With a limited amount of available energy, the total number of position recordings is limited. Therefore, a low-duty cycle has to be chosen for long-term operations, resulting in a low spatiotemporal resolution. Integrated energy harvesting, such as solar power, can extend the lifetime of a tracking device, which only holds for animals that are active in sunlight. Consequently, the design of a radio-tracking study on nocturnal animals such as bats is severely limited by this trade-off between the test campaign duration and measurement rate.

Bats can cover large distances between maternity roosts, foraging sites, and hibernation sites [11,12]. Looking at the highly maneuverable bats, the recommendation for an additional load by a tracking device is 5% of the animal’s body weight [13,14]. However, several short-term tracking studies have shown that some bat species of the family *Vespertilionidae* can carry higher percentages of additional loads without measurable impacts [15].

Our knowledge of bat movements, especially over long distances, depends almost entirely on other methods, such as bat banding. Recent studies have investigated bat migration, but there are massive gaps in our understanding of how bats migrate (e.g., [16,17]). The main reason for this is that a high-resolution tracking system that allows individual bats to be tracked with sufficient spatial and temporal resolution throughout the entire migration period is still lacking. The migration ecology of bats became a pressing issue for bat conservation, as it is known that migrating bats collide in large numbers with wind turbines in Europe and North America [18,19].

Many bat species show recurring spatiotemporal behaviors and return to the same places repeatedly (e.g., place of birth or hibernacula). In the context of the introduced tracking system, this behavior is important for planning the retrieval of the loggers and the data. Bat banding data from several decades showed that most recaptures are from the initial markings [20,21]. Females, especially, show a pronounced natal philopatry to their nursery roosts [22] and have a high rate of returning.

Here, we introduce a novel ultra-low power tracking system for small animals based on GSM (global system for mobile communications) cell logging. Compared to existing solutions, our system offers a significantly increased number of measurements and is suitable for long-term tracking over several months or for high-temporal-resolution tracking over several weeks. The system was applied with the greater mouse-eared bat, *Myotis myotis*, a species that covers long distances in daily movements, and with the long-distance migratory noctule bat, *Nyctalus noctula*. The survey areas offered easy access to the animals (e.g., bat boxes or maternity roosts in an attic), which allowed finding the animals to attach, read out, and later detach the loggers.

## 2. State of the Art

Several tracking systems for bats are commercially available or have been developed in academic research. VHF direction finding is a conventional approach for wildlife tracking and has been used on bats since the 1960s [23]. The miniaturized VHF transmitters periodically send uncoded CW signals in dedicated wildlife bands at 150 MHz in Europe and 166 MHz in North America. A transmitter design was described in [24]; it had a weight of 0.2 g, a runtime of 22 days, and output power of −21 dBm, giving a range of up to 2 km. Locating VHF transmitters is usually accomplished with hand-held directional antennas and a standard radio scanner that outputs the demodulated signal as short periodic beeps. For identification, some loggers are able to transmit an ID as Morse code, others operate on different channels. Depending on the chosen battery and duty cycle, runtimes range from days to multiple months, with the lightest weight starting from 0.2 g. Respective transmitters are offered by several companies. Reference [17] published a multi-hour mission on board a small aircraft, following individual bats in southern Germany, with an increased radius of action. However, due to the vast manual effort, VHF radio tracking is not appropriate for larger long-term and wide-range migration research campaigns.

The MOTUS network adds digitally modulated identifiers to the VHF signals and is able to identify and locate passing animals automatically [25]. Receivers with known positions simply conduct a presence detection. Directional sector antennas can narrow down the location accuracy at the cost of more hardware efforts or reduced coverage. MOTUS networks with automatic server backends are primarily operated in North America. However, coverage is far from being gapless, as a station’s range is only several hundred meters to ten kilometers, and base stations are put up by individual persons at their homes, institutes, or areas of interest. Transmitters have similar specifications as unmodulated VHF radio beacons [26].

The German research project BATS implemented an RF localization system with an accuracy in the range of meters, aimed for the investigation of flight trajectories and the group interaction of bats. The tags used two frequencies at 868 MHz and 2.4 GHz and achieved run-times of several days to a few weeks [27]. In addition, temperature, acceleration, barometric pressure, light levels, acoustic signals, and ECGs were successfully recorded and analyzed in several studies [28,29]. These data are not used for tracking but could provide additional insight into the behavior and physiology of bats. However, the coverage was limited to approximately 1 km${}^{3}$.

ATLAS, a localization system developed at the Israeli Minerva Center for Movement Ecology, was initiated in 2012 and is now operated in several countries worldwide [30,31,32]. It combines the advantages of BATS and MOTUS and provides high resolution and wide coverage. The tags are based on sub-1 GHz transmitters and transmit short FSK-modulated beacons with 1 MBit/s in the 433 MHz band. Due to their wide bandwidth and good autocorrelation properties, a localization accuracy of 5 m over distances of up to 15 km can be achieved with a time difference of arrival (TDoA) method. By using seven receivers, an area of 600 km${}^{2}$ can be covered, which could also be extended by setting up more stations [30]. Nevertheless, for truly unknown migrations, the coverage of ATLAS is still too low at a reasonable effort.

Since the end of the last century, global navigation satellite systems (GNSSs) have become available for civil use and they enable localization with globally consistent accuracy of a few meters. However, GNSS signals have very low power levels and can hardly be decoded within buildings or even forests without long acquisition times, as all GNSS almanacs must be available at the receiver. With so-called cold starts, a GNSS receiver has to decode the data from the signals, which typically requires 30 s with full-current consumption. In order to limit the consumed energy of wildlife GNSS devices, the reception is canceled after several seconds regardless of the success status—a so-called location attempt. Commercially available ultra-lightweight GPS loggers offer 130 fix attempts at 1.0 g [33]. GNSS loggers have been successfully used for the recording of velocity and height profiles of bat movements with high resolution but over very limited periods of time [34]. GNSS loggers do not have native data uplinks, so the animal’s position cannot be tracked remotely. Therefore, larger GNSS loggers (e.g., for birds) often come with a cellular module but at the cost of size, weight, and energy consumption, which does not fit the budget of a bat-carried logger.

ICARUS is a spaceborne telemetry system that is designed to provide a global and power-efficient data uplink and downlink for wildlife research applications [35,36]. Localization, however, is based on GNSS. Current modules have a weight of 5 g and a volume of 2 cm${}^{3}$, which is not yet feasible for deployment on bats. Nonetheless, tracking bats is a claimed future goal for ICARUS [37]. The space component was installed on the International Space Station in 2019 and started normal operations in 2020. However, it had to be suspended in March 2022 due to the end of international cooperation as a consequence of the Russian raid on Ukraine. [38].

Argos is another space-based localization and telemetry system: up to 30 satellites measure the Doppler frequency shifts of a transmitter signal from different orbits and calculate the transmitter’s position from the data. Argos, which started in 1978 and upgraded to a third generation in 2014, is widely applied, both as a search and rescue system in maritime and aviation applications, and for environmental and wildlife monitoring [39,40].

So-called geolocators use daylight and a clock with a calendar for localization. They measure light intensity over time due to Earth’s rotation and the obliquity of the ecliptic. Daylight, including day length and absolute time, is influenced by latitude, longitude, and the calendar day [41]. The principle is easy and very energy efficient, but the accuracy lies in the order of several hundred kilometers and depends on the geographical position. The Lotek FL6057, weighing 0.3 g [42], is one example of an ultra-lightweight device that can operate for over one year. However, due to this principle, only one localization per day can be acquired.

Flying bats can also be detected by radar. High-power radars (e.g., for maritime use) can resolve bats over several hundred meters [43]. Millimeter-wave radars have a short range but have been used successfully for detecting bats near wind turbines [44]. However, identifying individuals and distinguishing them from birds is difficult, making migration tracking impossible [45].

General-purpose localization methods could also be applied for tracking bats. Cellular networks have been widely deployed in the last 30 years and are predestined for locating purposes. Cellular localization principles can be distinguished between mobile-based, network-based, and hybrid principles. The cell ID method is a typical hybrid approach, where the mobile station scans all neighboring base stations and uploads that list to the network, which returns a coarse position to the mobile station (e.g., for speeding up an GNSS TTFF). Better accuracies can be accomplished with the time difference of arrival, which is applied from the network side for pinpointing a specific device [46]. However, commercial cellular modules are still too big and heavy for usage on small animals, such as bats [47].

Low-power wide-area networks (LPWAN), such as Sigfox or LoRaWAN, offer basic localization options based on RSSI measurements [48]. Improvements may be achieved with fingerprinting, TDoA, or angle estimation methods [49].

## 3. System Concept

The localization concept presented here is based on monitoring GSM cellular base stations, which periodically broadcast unencrypted cell identifiers (cell IDs) that are unique worldwide. By measuring the received power of the available cells, the location of the receiver can be estimated using a respective location database of cellular sites. Table 1 provides an overview of all current cellular standards and the requirements for a respective cell ID logger. As of 2023, 2G, 4G, and 5G are currently in operation, and could, in principle, be used for cell ID localization. However, as we had to develop an ultra-low power–miniaturized software-defined receiver, we decided to use 2G only, as the receiver complexity is low and, thus, the main goal of an energy-efficient implementation can be achieved most easily. GSM has been deployed almost worldwide since the 1990s and is today referred to as a second-generation (2G) network. UMTS (3G) was recently switched off in Germany in favor of frequency reuse with 4G and 5G networks; GSM is expected to remain in service (at least in Europe) due to its robustness and as a fallback for telephony [50].

Looking at Europe, GSM is historically operated in the 900 MHz and 1800 MHz bands, whereas the latter has been fully reallocated for LTE operation. Therefore, the main focus of our GSM logger is the reception of the 900 MHz band (B8) with a downlink frequency between 925 MHz and 960 MHz. In some areas in Germany, a 5 MHz wide LTE block is also operated within this band, which is detected and ignored by our logger. Since the GSM scanning is implemented in a software-defined radio receive-only approach, the logger requires no SIM card and is, hence, not limited to one network operator, increasing the number of receivable stations and minimizing the required energy by omitting transmit messages. By having no SIM card, our logger cannot connect to any cellular network—data uploads are not possible. The logger does not reveal its presence to the cellular operators as it does not transmit any GSM signals, which would be illegal anyway.

**Figure 1:**
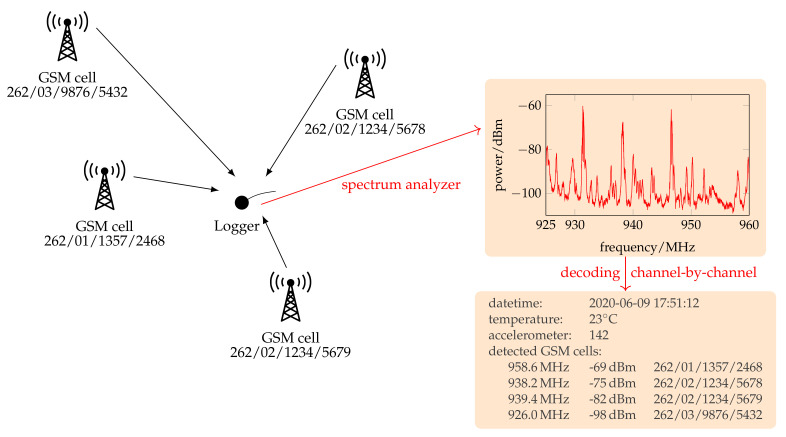
The logger receives four exemplary GSM cells with distinct cell IDs at different power levels. First, the spectrum of the GSM 900 band is analyzed, and up to 20 channels are chosen for decoding. The acquired data are then saved in the onboard memory.

Figure 1 shows an exemplary scan process during which a logger receives four GSM cells. The received power levels differ depending on the distance, terrain, and scenario. Theoretically, all 174 channels in band 8 could contain broadcast channels, but we limited the number of channels to be scanned to 20, sorted by signal power. The cell data are then stored to the onboard memory together with a timestamp, temperature, and an accelerometer value. That scanning process is repeated at an adjustable time interval during the mission. The position reconstruction is processed offline after regaining the logger (see Section 6).

## 4. Logger Hardware

**Figure 2:**
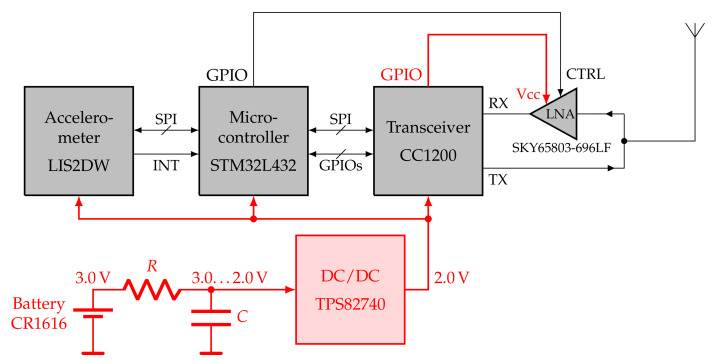
Block diagram of the miniaturized software-defined radio. The power supply is shown in red.

Integrated cellular chipsets are not yet available to low-volume customers as they used to be in the early 2000s. Instead, several suppliers have packaged cellular modules on the market that even provide commands for reading out cell IDs. However, no available module meets our requirements in terms of size, weight, and energy consumption. Therefore, we developed a miniaturized software-defined radio, specifically tailored to cell ID scanning. It is based on an integrated sub-GHz transceiver with an appropriate front end and antenna, a microcontroller, an accelerometer, and a power management unit (see Figure 2). The transceiver is meant to be used in general-purpose applications but is exploited by us for receiving GSM signals, which are demodulated and decoded on the microcontroller in real time [47]. The transceiver is also used for a bidirectional short-range packet communication mode in the European 868 MHz SRD band [47]. In the following subsections, the core components are described in detail.

### 4.1. Transceiver

**Figure 3:**
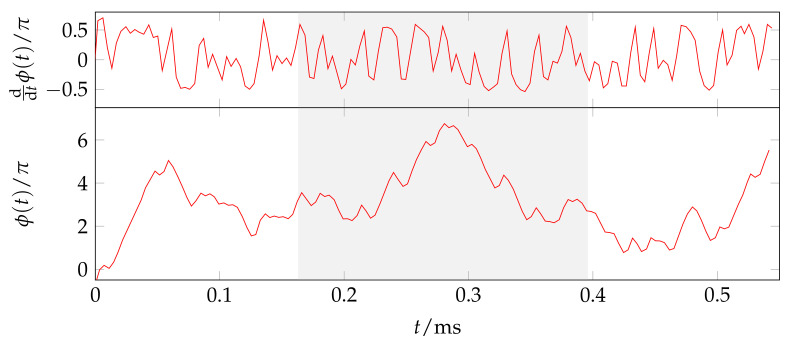
Instantaneous frequency $\frac{d}{dt}\phi \left(t\right)$ read-out from CC1200 (measurement) and absolute phase ϕ(t) calculated by integration. The plot shows a measured frequency and time-synchronized GSM SCH burst with its training sequence highlighted with a gray box.

The low-power sub-GHz transceiver CC1200 by Texas Instruments features a packet mode and supports the modulation schemes of frequency-shift keying (FSK) and on–off keying (OOK). In addition, it also provides documented access to baseband signals in the receiver chain via registers. As both FSK and GMSK are phase-continuous modulation schemes, we use the internal register CFM_RX_DATA_OUT, which gives time-synchronized samples of the instantaneous frequency $\frac{d}{dt}\phi \left(t\right)$ from the FSK demodulator block for the acquisition of GSM baseband signals [47]. However, for the demodulation of the GMSK-modulated GSM signals, the absolute phase ϕ(t) is required. It is calculated by integration and frequency error compensation (see Figure 3) [47].

An RF matching network for the differential receiver’s input and the single-ended power amplifier’s (PA) output is realized with several discrete inductors and capacitors and an additional low-noise amplifier (LNA) for improved receiver sensitivity. Further insights into the development of the RF network can be read in [47]. With the CC1200, we implemented an appropriate analog and digital front-end for the GSM downlink band, including band and channel filtering, a down-conversion, and analog-to-digital conversion, at a current consumption of only 23 mA during receiving and at a small form factor of 5 mm × 5 mm. However, with this solution, the receiver bandwidth is limited to 200 kHz, which is only sufficient for 2G signals (see Table 1).

### 4.2. Microcontroller

The STM32L432 ARM Cortex-M4 microcontroller also comes in a 5 mm × 5 mm QFN package and is the central logic element, which controls the transceiver and the accelerometer, accomplishes the GSM signal processing, controls the overall program sequence, and stores the obtained data in its onboard flash memory. Insights into selected signal processing optimizations can be gained from [47].

The STM32L432 has several operating modes, which are dynamically selected by our application. Compared to a solution with a dedicated DSP, the microcontroller provides high flexibility for task-wise optimization between the clock rate and power consumption. Most of the time, the microcontroller is on standby with the real-time clock (RTC) running, which consumes 280 nA. For processing GSM signals in real time, the core is clocked at its maximum frequency of 80 MHz, which results in a current consumption of 11 mA.

### 4.3. Power Supply

**Figure 4:**
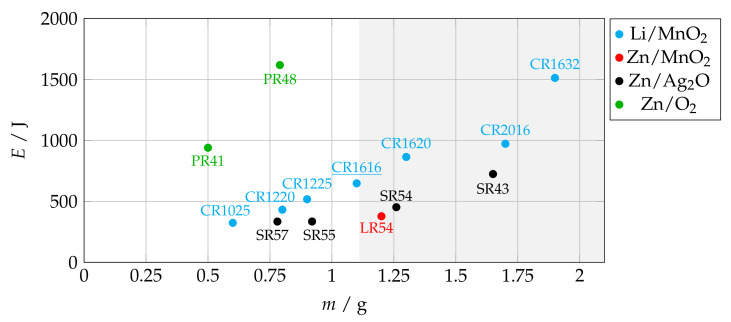
Mass and energy of commercially available coin cell batteries by technology.

The logger is powered by a lithium manganese dioxide (Li/MnO${}_{2}$) battery of type CR1616 with a nominal voltage of 3 V and capacity of 60 mAh. The cell has the highest energy for an acceptable mass m≤1.1 g. Zinc–air batteries have a higher specific energy, but they require an inlet for air, which is not possible with a hermetically sealed logger (see Figure 4).

**Figure 5:**
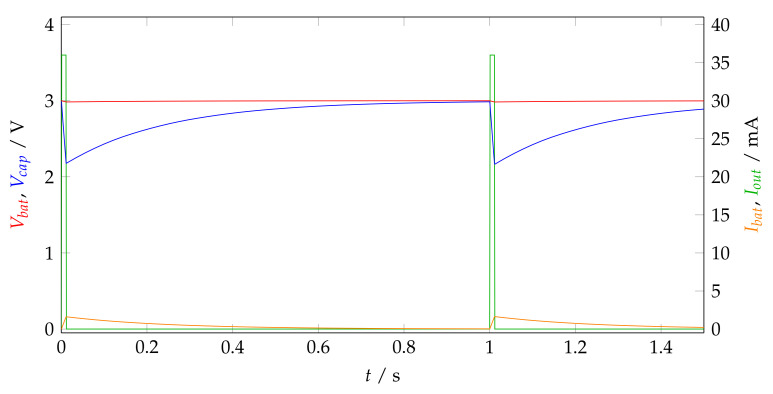
Simulated battery and capacitor voltages and currents [47].

A buck converter (TPS82740) supplies the components with a voltage of 2.0 V. Small coin cells, such as the ones chosen, cannot deliver high peak currents due to the relatively high internal resistance. Therefore, the entire software functionality is designed on the following premise: no operation may take longer than 15 ms, during which, a large capacitor buffering the supply voltage feeds the step-down DC/DC converter (see Figure 2). The discharge time must be followed by a recharge time of at least 1 s, during which all active components must be on standby. Figure 5 depicts the duty cycling of the power supply.

### 4.4. Mechanical Design

**Figure 6:**
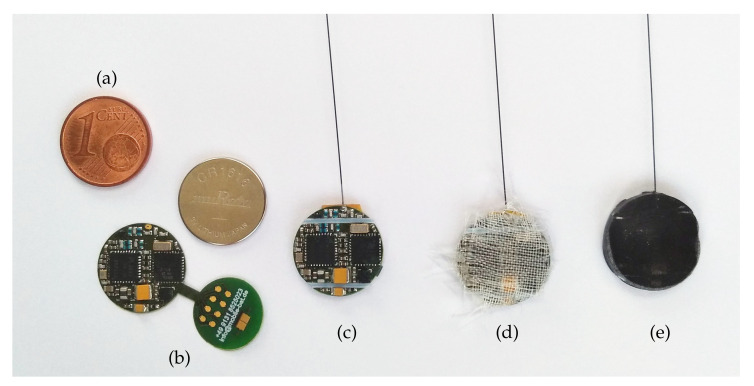
Manufacturing steps of the miniature GSM cell logger: (**a**) the 1 euro cent coin as a size reference, (**b**) assembled printed circuit board and CR1616 coin cell, (**c**) folded around the battery, (**d**) with fiberglass fabric, (**e**) molded in epoxy resin. The antenna has a length of 8 cm and is not fully visible [47].

All components are assembled on a 25 µm-thin flexible printed circuit board (PCB) on the top side. The PCB consists of two round interconnected shapes with the exact diameter of the coin cell, allowing them to be bent around the battery and make contact with both poles (see Figure 6). On the fan-out, there are pads for the serial wire debug (SWD) and universal asynchronous receiver–transmitter (UART) for debugging purposes, as well as a pad, in series with the battery that needs to be connected in order to start up the logger.

Since the loggers must withstand rain, mechanical strains such as scratching or biting, and exposure to caustic excrement over several months, the battery and electronics are encapsulated in fiber-reinforced resin. In the first experiments without glass fiber fabrics, the epoxy resin rubbed off at the edges and became leaky. The subsequently introduced fiberglass fabric adds only 30 mg of weight but improves durability significantly. In order to achieve a reproducible shape, silicone molds were used for casting.

In several short-term tests, the loggers were glued to bats and fell off again, such as intended after 2–3 weeks. However, for long-term mounting lasting up to one year, an additional fixture system is implemented: Two tubes with an inner diameter of 0.5 mm and an outer diameter of 0.75 mm are arranged between the electronic components and seamlessly molded into the epoxy casing. A durable suture is fed transversely through them and is sewn lengthwise into the animal’s epidermis.

**Figure 7:**
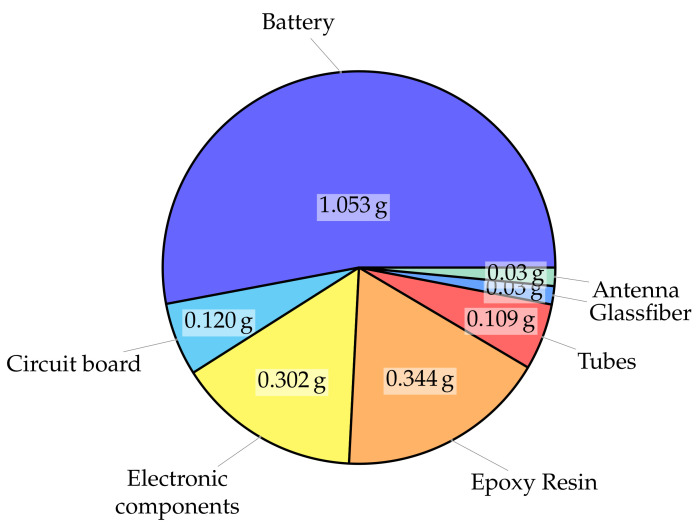
Weight contributions of the logger by components. Total weight: 1.988 g [47].

When looking at the logger’s weight, the battery contributes 1.053 g. All electronic components amount to 0.302 g, and the circuit board weighs 0.120 g. The two tubes of the mounting system add 109 mg and the fiberglass fabric adds another 30 mg. A slight variability in additional weight is contributed by the epoxy in which the logger structure is embedded. In all, about 50% of the total mass is for energy supply, about 25% is for the electronics, and about 25% is for the mechanical sealing. A diagram of the weight contributions is shown in Figure 7.

### 4.5. Energy Characterization

The logger’s energy consumption is characterized and summarized in Table 2. Most of the time, the circuit is in standby mode, during which it records accelerometer events and waits for the internal alarm to trigger a new measurement. This mode consumes 1.5 µA, resulting in energy consumption of 388.8 mJ over a 24 h period. A spectrum scan takes 361 ms and consumes 30 mA or 32.5 mJ per scan. From the resulting spectrum, up to 20 channels are selected for decoding. Each cell may take a different period of time for decoding, as the receiver is first unsynchronized and starts with a random position in the GSM frame structure. Furthermore, retrials, in case of decoding failures or after time outs, will add to the processing time for the respective cell. In the best case, a single cell decoding consumes 0.9 mJ, if the receiver was initially switched on at exactly the right GSM timeslot. An average cell decoding, however, requires 32 ms due to synchronization and amounts to 2.9 mJ. A complete GSM measurement over all cells consumes 86.9 mJ (see Table 2).

The RF TRX detection signal that is transmitted by the logger once a day requires 9.3 mJ per day, if no RF TRX base station answers. That is the normal case if the logger is out of range of an RF TRX base station. However, if a connection is established and the RF TRX base station requests a full data download from the logger, a maximum energy of 78.3 mJ is required. The logger stops the download after no longer than one minute in order to limit its own energy consumption.

## 5. RF TRX System for Return Detection and Data Upload

**Figure 8:**
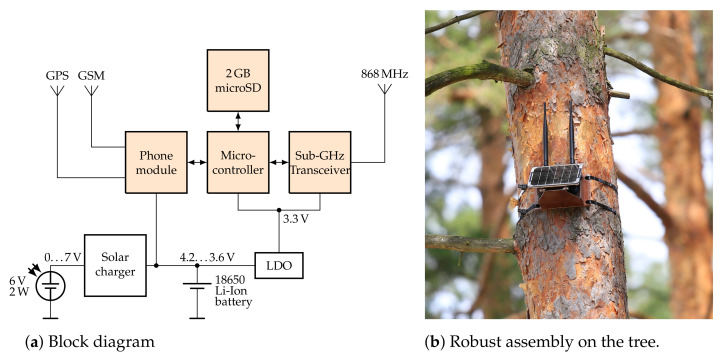
RF TRX base station for return detection and data upload. (**a**) The phone module (FONA 808) is directly supplied from the li-ion battery due to high peak currents during TX, whereas all other components are operated at 3.3 V. (**b**) Solar-powered base station mounted at a tree. The left antenna is for the wireless sensor network in the 868 MHz band, and the right antenna is used for the GSM/GPRS phone connection. On top of the box is a GPS antenna (not visible). The waterproof aluminum box is mounted to a tree with long zip ties [47].

Re-gaining the loggers after one year is a crucial problem with high uncertainty: it is only if the stored data can be read out that the bat’s positions over the last months can be reconstructed. During the absence of the animal, its whereabouts are unknown. It is known, however, that the animal cyclically returns home to its maternity roost, e.g., the place where the logger had initially been attached. Therefore, a local radio system for return detection and data upload has been set up at that place for the detection of returned bats and for wirelessly downloading the acquired data (Figure 8). In order to be as energy-efficient as possible for the logger, the radio schedule has been defined according to the bat’s nocturnal activity. The bat may change locations within the local forest during darkness, but it spends the day at a place. A network of our TRX systems has been deployed at eight distant boxes. At approximately 8:00 in the morning, the logger transmits a short beacon that is eventually detected and answered by one TRX base station. Each logger has a time slot of one minute at its disposal during which it can also upload its gathered data. In case the logger’s real-time clock (RTC) drifts apart or is unset, and the beacon is transmitted at the wrong time, the base station monitors the band 24/7 and sets the logger’s RTC correctly once a connection is established. At 9:00 every morning, the base station enables its mobile phone module, connects to a web server, and uploads a status report, and all data are downloaded from the loggers into an SQL database. The server then sends an email to the authors, offering them ample time to look for the detected bat before sunset. To cover a larger area, several of the boxes can be set up since they synchronize their downloaded data over the server. This prevents different boxes from downloading the same data from the loggers several times as the animals move between them. More details about the TRX system can be found in [51].

The TRX base station hardware is powered by a solar panel that recharges a lithium battery of type 18650 and can supply it for a period of one week without sunshine in full-receive mode (I=25mA). Below 3.8 V, the receiver enters a duty-cycle mode, in which two out of five beacons can still be detected. This power-saving mode can be operated for an additional two weeks without direct sunlight. Below a voltage of 3.6 V, the system shuts down in a defined manner in order to avoid under-voltage, as that turns out to cause irreversible damage to the memory card file system. With enough sunlight, the TRX base station wakes up from hibernation mode and resumes normal operation. A network of seven TRX stations operated between 09/2019 and 08/2021, without any failures, ultimately playing a decisive role in the success of the project by detecting the return of one bat after half a year.

## 6. Position Reconstruction

The position of each recorded cell ID measurement is estimated using an RF pattern-matching approach (also known as RF fingerprinting). The recorded power levels of each cell, the so-called signatures, are compared with a database of respective propagation simulations. First, the propagation of every cell is computed once. Their geographical coordinates, height above ground, azimuth direction, and transmit power were kindly provided by all three German cellular operators, as per our request. Afterward, position estimations based on the simulations and a mathematical model were conducted for each measurement [47].

### 6.1. Propagation Simulation

**Figure 9:**
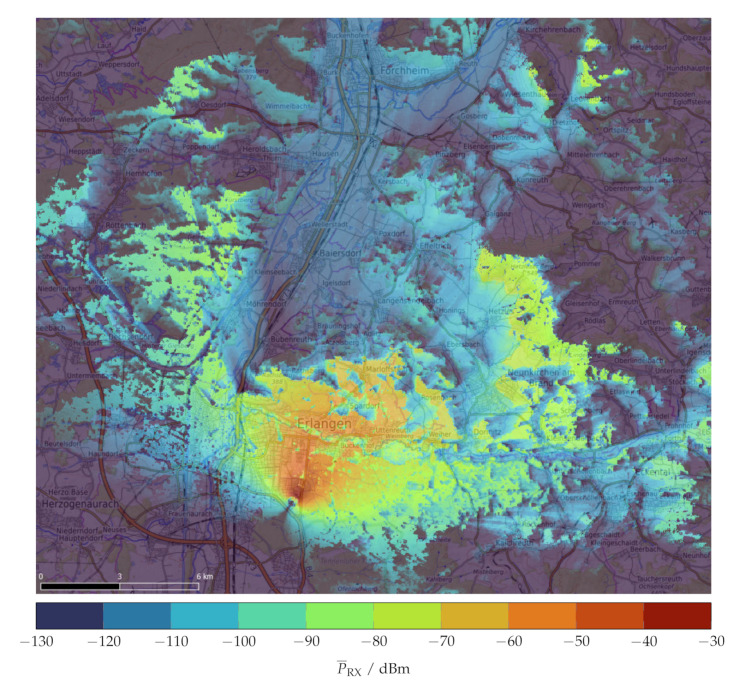
Predicted mean receive power ${\bar{P}}_{\mathrm{RX}}$ of an isotropic receiver at $h_{\mathrm{RX}}=2$ m for GSM cell 262/03/58162/8863 (N49.573859/E11.027138, $h_{\mathrm{RX}}=52$ m, $\theta =30^{\circ }$) [47].

The Longley–Rice model is a generic propagation model that describes the statistical attenuation in an irregular terrain. It is one of the most important standard propagation models for VHF and UHF frequencies. The model includes TX and RX coordinates, frequency, TX and RX antenna heights above ground, polarization, ground properties, and statistical parameters. The Longley–Rice model features a point-to-point mode, which can include a digital terrain model. By calculating the loss for each point on a map, a 2D attenuation map is generated. We used the open-source software “SPLAT”, which implements the Longley–Rice model and calculates a predicted receive power map for a circular area of 35 km around each cellular base station. Figure 9 depicts a receive power map for an isotropic receiver at 2 m above ground for every position around a base transceiver station. Every propagation prediction has a pixel size of 30 m × 30 m and a pixel value for the simulated mean receive power ${\bar{P}}_{\mathrm{RX}}$. These predictions were saved as georeferenced GeoTIFF files, allowing for easy handling with GDAL tools and integration into a GIS [47].

### 6.2. Position Estimation

The mathematical basis of the position estimator relies on the conditional probability *P*, with event *A* representing the “receiver is at this position” and event $B_{i}$ representing “the receiver measured the power $P_{i}$ of base station *i*”:   

(1) $$P\left(A\;|\;B_{1}\cap B_{2}\cap \ldots \cap B_{N}\right)=P\left(A|\overset{N}{\underset{i=1}{⋂}}B_{i}\right)$$

Under the assumption that all propagation channels are uncorrelated and with the Bayes’ theorem, the probability *P* for a location *A* under the condition of all events $B_{i}$ can be written as:

(2) $$\begin{array}{cc} P\left(A|\overset{N}{\underset{i=1}{⋂}}B_{i}\right) & =\frac{P\left(\overset{N}{\underset{i=1}{⋂}}B_{i}|A\right)\times P\left(A\right)}{P\left(\overset{N}{\underset{i=1}{⋂}}B_{i}\right)}=\frac{P\left(B_{1}|A\right)\times P\left(B_{2}|A\right)\times \ldots \times P\left(B_{N}|A\right)\times P\left(A\right)}{P\left(B_{1}\right)\times P\left(B_{2}\right)\times \ldots \times P\left(B_{N}\right)}=\\ \; & =\frac{\prod_{i=1}^{N}P\left(B_{i}|A\right)}{\prod_{i=1}^{N}P\left(B_{i}\right)}\times P\left(A\right)=\prod_{i=1}^{N}\frac{P(B_{i}|A)}{P(B_{i})}\times P\left(A\right) \end{array}$$

The amplitude of a signal in a propagation channel without direct sight is Rayleigh-distributed. Its probability density function f(a) can be written as

(3) $$f\left(a\right)=\frac{a}{\sigma^{2}}\times e^{-\frac{a^{2}}{2\sigma^{2}}}$$

With the substitution $P_{\mathrm{RX}}=a^{2}$, the density transforms to

(4) $$f\left(P_{\mathrm{RX}}\right)=\frac{1}{\sigma^{2}}\times e^{-\frac{P_{\mathrm{RX}}}{\sigma^{2}}}=\frac{1}{{\bar{P}}_{\mathrm{RX}}}\times e^{-\frac{P_{\mathrm{RX}}}{{\bar{P}}_{\mathrm{RX}}}}=\underset{\mathrm{exponential}\;\mathrm{distribution}}{\underset{︸}{\frac{1}{\mu }\times e^{-\frac{x}{\mu }}}}$$

The mean receive power ${\bar{P}}_{\mathrm{RX}}$ corresponds to the root mean square of the Rayleigh distribution $\sigma^{2}$. From this, it can be concluded that the power distribution of a Rayleigh-distributed amplitude signal is identical to an exponential distribution with $\mu ={\bar{P}}_{\mathrm{RX}}$.

This gives the distribution function $F(P_{\mathrm{RX}})$

(5) $$F\left(P_{\mathrm{RX}}\right)=\int_{0}^{P_{\mathrm{RX}}}f\left(P_{\mathrm{RX}}^{\prime }\right)dP_{\mathrm{RX}}^{\prime }=1-e^{-\frac{P_{\mathrm{RX}}}{{\bar{P}}_{\mathrm{RX}}}}$$

The probability that the measured power $P_{\mathrm{RX}}$ is within the interval between $P_{\mathrm{RX},1}$ and $P_{\mathrm{RX},2}$ is the difference between both probabilities:

(6) $$P\left(P_{\mathrm{RX},1},P_{\mathrm{RX},2},{\bar{P}}_{\mathrm{RX}}\right)=\left(1-e^{-\frac{P_{\mathrm{RX},2}}{{\bar{P}}_{\mathrm{RX}}}}\right)-\left(1-e^{-\frac{P_{\mathrm{RX},1}}{{\bar{P}}_{\mathrm{RX}}}}\right)=e^{-\frac{P_{\mathrm{RX},1}}{{\bar{P}}_{\mathrm{RX}}}}-e^{-\frac{P_{\mathrm{RX},2}}{{\bar{P}}_{\mathrm{RX}}}}$$

With the power discretized in 1 dB steps, the probability *P* for a measured receive power $P_{\mathrm{RX}}$ in a 1 dB interval, i.e., $P_{\mathrm{RX}}\pm 0.5$ dB, and the simulated mean power ${\bar{P}}_{\mathrm{RX}}$ is:

(7) $$P\left(P_{\mathrm{RX}},{\bar{P}}_{\mathrm{RX}}\right)=e^{-\frac{a\times P_{\mathrm{RX}}}{{\bar{P}}_{\mathrm{RX}}}}-e^{-\frac{P_{\mathrm{RX}}}{a\times {\bar{P}}_{\mathrm{RX}}}}\;\mathrm{with}\;a=10^{0.5/10}\approx 1.122$$

**Figure 10:**
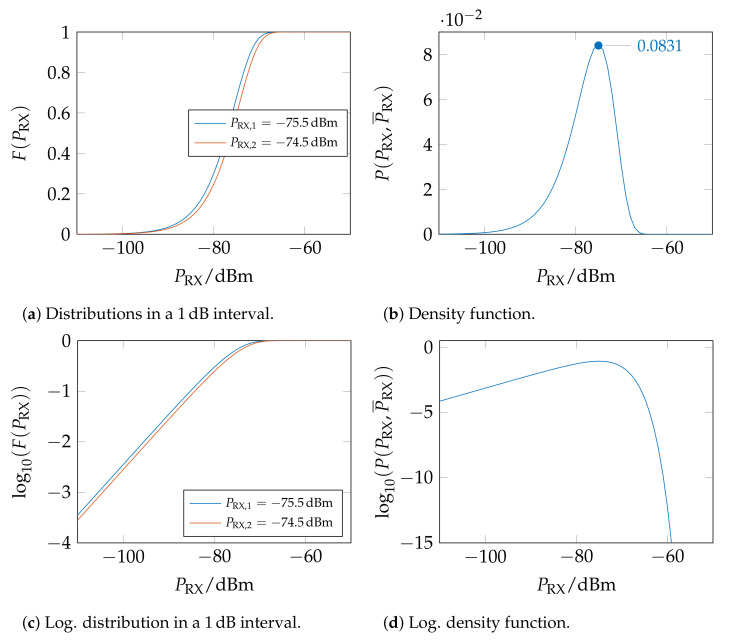
Example of the distribution for $P_{\mathrm{RX},1}=-75.5$ dBm and $P_{\mathrm{RX},2}=-74.5$ dBm and density function for a received signal with $P_{\mathrm{RX}}=-75$ dBm in a 1 dB interval [47].

The respective distributions and densities are plotted in Figure 10. For simplification, a logarithmic likelihood $P^{\prime }$ is calculated by normalizing the probability to its maximum:

(8) $$\begin{array}{cc} P^{\prime }\left(A|P_{\mathrm{RX}},{\bar{P}}_{\mathrm{RX}}\right) & =\log_{10}\left(\frac{1}{\max\left(P(P_{\mathrm{RX}},{\bar{P}}_{\mathrm{RX}})\right)}\times P\left(A|P_{\mathrm{RX}},{\bar{P}}_{\mathrm{RX}}\right)\right)\approx \end{array}$$

(9) $$\begin{array}{cc} \; & \approx \log_{10}\left(\frac{1}{0.0831}\times P\left(A|P_{\mathrm{RX}},{\bar{P}}_{\mathrm{RX}}\right)\right) \end{array}$$

The combined logarithmic likelihood $P^{\prime }$ for all measured and simulated received power levels can be calculated from the sum of all logarithmic likelihoods:

(10) $$P^{\prime }\left(A|\overset{N}{\underset{i=1}{⋂}}P_{\mathrm{RX},i}\right)=\sum_{i=1}^{N}P^{\prime }\left(A|P_{\mathrm{RX},i},{\bar{P}}_{\mathrm{RX},i}\right)$$

**Figure 11:**
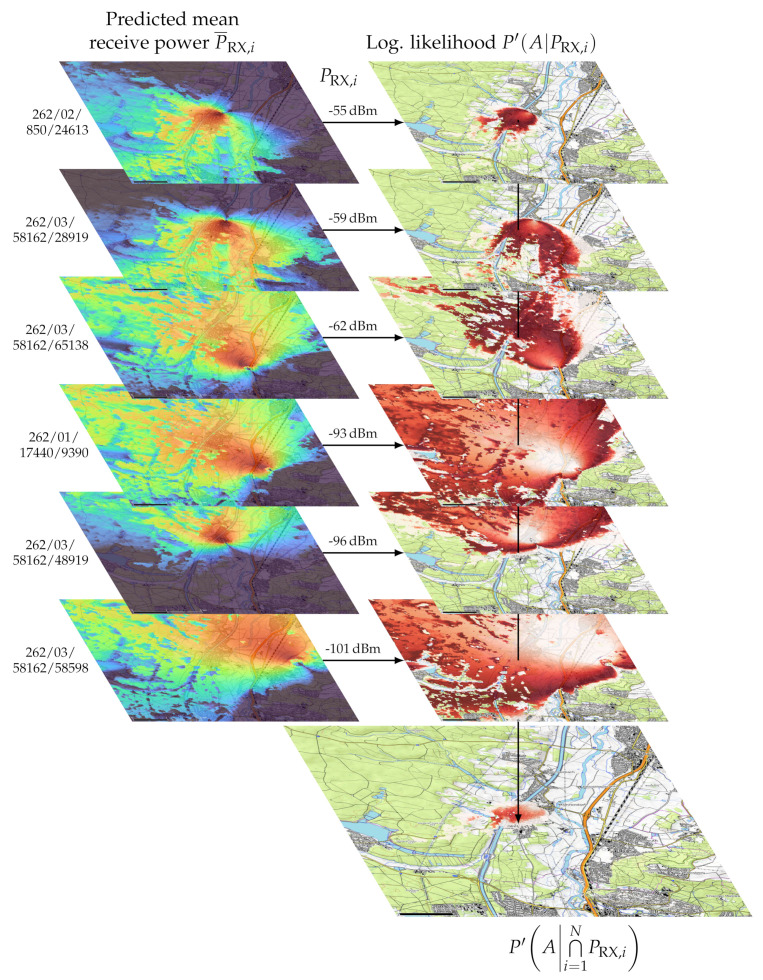
Depiction of the position estimation showing a reference measurement. The true position is in the center (base map: © OpenTopoMap) [47].

This computation was accomplished on the georeferenced raster images with the program gdal_calc.py from GDAL tools, which outputs one georeferenced likelihood raster image. Figure 11 shows the position estimation for an example measurement [47].

### 6.3. Accuracy

The achievable accuracy of the implemented Bayesian estimation shows a wide range and depends on multiple factors, for example:

- Number of base stations: With only one base station acquired, the receiver is in a circular ring around that base station. The more base stations that are decoded, the smaller the most probable area becomes. However, faulty cells (e.g., wrong cells in the database) may lower the accuracy.
- Power levels: High receive power levels (e.g., −60 dBm…−40 dBm) occur in a small area around the respective cell. The area increases proportionally to the square of the distance.
- Height above ground: The actual height of the receiver is unknown. Therefore, our propagation simulations were done for typical flight heights of the bats (2 m, 6 m, 18 m, 40 m).
- Terrain: Mountains and valleys have a high influence on signal propagation. We included a digital elevation model in our propagation simulations, so shadowing effects reduce the covered area and, thus, contribute to higher accuracy.

We conducted a coarse characterization of the system’s accuracy at different reference positions. We found values of 100 m in a city and 5 km for rural sites. However, it is not possible to derive a well-estimated accuracy, such as the dilution of precision values of GNSS receivers.

## 7. System Validation

The system was validated in several field tests: In a short-term test that took place over 2 weeks, 21 greater mouse-eared bats (*Myotis myotis*) were equipped with loggers at a bat research site in Ahrbrück, Germany. In July, the bats fly out eventually at night but always return to the site. Therefore, radio access was given and the position data could be downloaded via the TRX system on a daily basis. The loggers were programmed for a GSM measurement every ten minutes, which theoretically allowed about three weeks of runtime in terms of memory and battery. Bats were caught in a roost site, checked for health conditions, measured, tagged, and released at the same place after approximately 10 min of handling. The loggers were attached with surgical glue (Sauer Hautkleber, Manfred Sauer GmbH). Overall, 14,872 GSM localizations with a total of 59,566 decoded GSM cells were successfully recorded and a total run-time of 3801 h was achieved. The best logger recorded 1373 localizations within 322 h (see Table 3). The field test ended with the loggers falling off or being removed. The reconstructed travel routes are not part of this article and will be published separately.

A long-term field test for tracking bat migration, which took place over several months, was conducted at Havelberg/Elbe in 2019/2020. The loggers were programmed to do four measurements per day, at 12:00, 22:00, 0:00, and 02:00. A total of 48 loggers were sewn at the backs of female bats. The fixture system turned was insufficient and most animals were found months later in good condition, but without loggers. Only one animal with an attached logger was recaptured successfully after 223 days. With our system, both the migration route and the hibernation site could be precisely reconstructed. These biogeographical data will also be published separately. Reference loggers that were not attached to bats demonstrated a fully operational runtime of 11.2 months, during which they successfully conducted four measurements per day. After that, the logger still transmitted its TRX signal once a day.

## 8. Discussion

Looking at current bat-tracking systems, a basic distinction can be made between remote localization systems that require a custom ground infrastructure and self-localization systems that are not dependent on custom infrastructure (see Table 4).

Remote localization systems require the animal’s whereabouts to be roughly known beforehand, as a certain ground infrastructure has to be installed at suitable places before. If an animal, however, leaves the covered area, there is no chance to gain knowledge about its position. In contrast, self-localization systems make use of signals of opportunity (SOOP), i.e., signals from the environment that carry known information; these may be GNSS signals, cellular signals, any other radio signals, or just daylight and a clock. The advantage is that the covered area is defined by the coverage of the respective SOOP.

Table 4 shows the specifications of the systems described in Section 2. Most competing systems are GNSS loggers, which are easy to use in terms of data evaluation. However, our system can achieve a much higher number of localizations and is, therefore, suitable to track movement routes with high temporal resolutions over extended periods of time. The accuracy of GNSS is superior and more consistent with our cell ID approach, but only if GNSS signals are receivable. There are many scenarios when GNSS fails (e.g., inside tree holes, buildings, under bridges), where GSM coverage is instead perfectly available. GNSS loggers output geographical coordinates with high precision, our solution requires a complex position reconstruction from the cell IDs to coordinates, and the accuracy varies with base station density, terrain, and vegetation, and ranges from 100 m to 5 km in our test areas. This is completely sufficient, at least for tracking flight routes over larger distances, where a higher temporal resolution might deliver additional insights into the departure and arrival times and average airspeed. The cellular base station density typically depends on population density, where there are more cells in urban areas than in rural areas. The position accuracy of our system decreases with the lower number of decoded cell IDs as well.

## 9. Conclusions

We introduced a novel cellular tracking system for lightweight nocturnal animals that achieved an unprecedented runtime of 11.2 months. The loggers weigh 2.0 g, including the battery, and are capable of autonomously monitoring and decoding GSM broadcast signals. Compared to other wildlife tracking systems, our concept of a passive logger does not require any additional infrastructure besides being within GSM coverage. This coverage is available almost everywhere in Europe and many other countries. Compared to GNSS loggers, our logger allows for an order of magnitude larger number of logged locations. It is, thus, the very first bat-tracking system capable of seamlessly tracking an annual migration cycle.

## Figures and Tables

**Table 1:** Comparison of cellular standards by requirements for cell ID logging.

	2G (GSM)	3G (UMTS)	4G (LTE)	5G (NR)
Channel bandwidth ${}^{*1}$	200 kHz	5 MHz	1.4 MHz	3.6 MHz ${}^{*2}$
Modulation	GMSK	QPSK + DSSS	QPSK + OFDM	QPSK + OFDM
Frequency bands ${}^{*3}$	900 MHz	–	700/800/900/1800 MHz2.1/2.6/3.6 GHz	700/1800 MHz2.1/3.6 GHz
Receiver complexity	low	high	high	high

${}^{*1}$ for physical broadcast channels carrying cell ID information; ${}^{*2}$ for the 15 kHz subcarrier spacing; ${}^{*3}$ band usage as of 2023 in Germany, to the authors’ best knowledge.

**Table 2:** Measured logger current consumption by modes. The CR1616 battery provides nominal energy of 648 J.

Mode	Operation	Current	Duration	Energy	Condition
Standby	RTC & ACC	1.5 µA	∼24 h	388.8 mJ	per day
GSM	spectrum scan	30 mA	361 ms	32.5 mJ	per scan
	cell decoding	30 mA	32 ms	2.9 mJ	per cell (best case: 0.9 mJ)
	total	%	%	86.9 mJ	per complete GSM measurement ${}^{1}$
RF TRX	detection signal	29 mA	107 ms	9.3 mJ	per day (base station not in range)
	data upload	29 mA	900 ms	78.3 mJ	per day (full usage of all timeslots)

${}^{1}$ 10 decoded cells.

**Table 3:** Acquired data during a short-term field test in Ahrbrück/Germany in 07/2019. A total of 21 loggers were deployed.

	Avg	Best	Total
localizations	708.2	1373	14,872
decoded base stations	2836.5	5590	59,566
run-time/days	7.5	13.4	158.3
memory size	7.5 kB	167.1 kB	1.7 MB

**Table 4:** Comparison of small animal-tracking systems split into remote localization and self-localization systems.

Category	Remote Localization				Self-Localization		
System	VHF transmitter	MOTUS (coded VHF)	BATS	ATLAS	Geolocator	GNSS logger	Mobile-BAT
Source	[52]	[25,26]	[53]	[30,32]	[41,42]	[33]	this work [47]
Method	radio beacon	presence detection, AoA	RSS-DoA	TDoA	daylight + clock	GNSS	GSM cell ID
Required infrastructure	direction finding equipment	stationary receivers	stationary receivers	stationary receivers	none	none	none
Frequency	150/166 MHz	150/166 MHz	868/915 MHz + 2.4 GHz	433 MHz	–	1.5 GHz	900 MHz
Weight	from 0.2 g	from 0.2 g	1 g	1 g …10 g	0.3 g	1.5 g	2.0 g
Runtime	days to months	days to months	days to weeks	days to weeks	12 months	days to weeks	11.2 months
Number of localizations	depends on human resources	depends on detects	350,000 ${}^{*1}$	100,000 …1 M	365 (once per day)	up to 130	1534
Coverage	depends on human resources	∼10 km^2^ per station	∼1 km^2^	∼1000 km^2^	global	global	global (where GSM coverage)
Accuracy	depends on human resources	100 m …10 km	4 m	5 m	up to 23 km ${}^{*2}$	2 m	100 m …5 km

${}^{*1}$ 30/min for 8 days. ${}^{*2}$ Various for latitude and longitude.

## Data Availability

The data presented in this study are available on request from the corresponding author.

## Abbreviations

The following abbreviations are used in this manuscript:

ACC	accelerometer
AoA	angle of arrival
DFG	German Research Foundation
ECG	electrocardiography
FSK	frequency-shift keying
GIS	geographic information system
GMSK	Gaussian minimum-shift keying
GNSS	global navigation satellite system
GPRS	general packet radio service
GPS	global positioning system
GSM	global system for mobile communication
LNA	low-noise amplifier
LPWAN	low-power wide-area network
LTE	long-term evolution
MLS	Mozilla location service
PCB	printed circuit board
PTFE	polytetrafluoroethylene
QFN	quad flat no-leads package
RF	radio frequency
RTC	real-time clock
RX	receive
SOOP	signals of opportunity
TDoA	time difference of arrival
TRX	transmit and receive
TTFF	time to first fix
TX	transmit
VHF	very high frequency
